# Scottish Seaweeds as Sustainable Sources of Micronutrients: Mineral Composition, Vitamin B_12_ Content, and Safety Assessment

**DOI:** 10.1002/fsn3.71988

**Published:** 2026-06-04

**Authors:** Ibrahim Almosa, Shabina Bashir, Alan A. Sneddon

**Affiliations:** ^1^ The Rowett Institute University of Aberdeen Aberdeen UK

**Keywords:** blanching, heavy metals, iodine, macroalgae, macrominerals, micronutrients, nutrients, seaweed, sustainable food, vitamin B_12_

## Abstract

Seaweeds (macroalgae) are increasingly recognized as potential nutrient‐dense components of human diets and promising contributors to sustainable food systems. The unique nutritional profile, along with a rich content of soluble fiber and bioactives, has led to growing interest in the potential of seaweeds in addressing micronutrient insufficiencies, particularly in plant‐based diets where traditional sources of certain micronutrients are limited. This study aimed to evaluate selected Scottish seaweed species by quantifying key micronutrients and estimating their potential contribution to dietary intakes while also determining heavy metal and iodine concentrations to assess consumer exposure risk. In addition, the study investigated practical mitigation strategies to reduce iodine and heavy metal levels, with the objective of delivering nutritionally valuable seaweed products that are both safe and aligned with dietary guidelines. Eleven wild seaweed species obtained from the north coast of Scotland together with two farmed seaweed species from the west coast were analyzed for mineral and heavy metal content using inductively coupled plasma–mass spectrometry (ICP‐MS), and vitamin B_12_ content by an enzyme‐linked immunosorbent assay. Seaweed iodine and heavy metal content were used to assess potential health risks. Additionally, some seaweed species were subjected to a blanching process in water heated at different temperatures to assess the effects on reducing seaweed iodine and heavy metal content. All wild and farmed Scottish seaweeds contained detectable levels of vitamin B_12_, with the highest content observed in *Fucus* species, with *F. serratus* showing a mean value of 148.0 ± 42.5 μg/kg dry weight. This was estimated to potentially provide 49% of the RNI for vitamin B_12_ in a 5 g portion. Among the minerals analyzed, a 5 g portion of seaweed was estimated to provide notable amounts of the RNI for potassium (up to 18%), magnesium and calcium (around 10%), and iron (up to 83%), but these were dependent on seaweed species or whether farmed or not. Most seaweeds, however, contained high iodine levels that were estimated to pose a risk to health. Blanching of *A. esculenta* , *S. latissima*, and *F. serratus* for 5 min at 80°C showed that seaweed iodine content could be reduced by around up to 90% in all three species, while in the latter two species it additionally reduced levels of arsenic by 39% and 52%, respectively. Scottish seaweeds show strong potential as sustainable sources of essential micronutrients, particularly for populations who do not consume animal products, such as vegetarians. However, the bioavailability of vitamin B_12_ was not assessed in this study and should be examined in future work. In addition, heavy metal concentrations must remain within safe limits to minimize potential health risks. Therefore, future studies should focus on optimizing harvesting and processing methods to ensure health risks are minimized while the nutritional benefits are optimized.

## Graphical abstract

A schematic outline of the study showing where seaweeds were harvested, how they were analyzed and then evaluated for micronutrients.

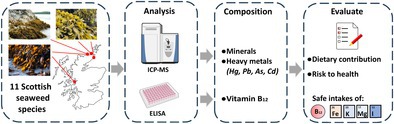

## 1 Introduction

Seaweeds (marine macroalgae) are increasingly recognized as nutrient‐dense components of human diets and promising contributors to sustainable food systems. Unlike most terrestrial vegetables, seaweeds accumulate a broad spectrum of minerals and micronutrients directly from seawater, often at concentrations substantially higher than those found in land plants, including essential trace elements such as iron, zinc, magnesium, and potassium (Peñalver et al. 2020; Lomartire et al. 2021; Aakre et al. 2021). This unique nutritional profile, along with their rich content of soluble fibers and bioactive compounds, has led to growing interest in their potential health benefits and role in addressing micronutrient insufficiencies, particularly in plant‐based diets where traditional sources of certain micronutrients are limited (Lomartire et al. 2021; Aakre et al. 2021).

One micronutrient of particular interest is vitamin B_12_ (cobalamin), a nutrient typically obtained from animal‐derived foods. While reliable plant sources of bioavailable vitamin B_12_ are rare, some seaweeds, particularly red algae such as Porphyra (nori), have been reported to contain appreciable amounts of this vitamin, raising the possibility that seaweeds could contribute to dietary B_12_ intake (Peñalver et al. 2020; Cherry et al. 2019). However, reported levels can vary widely between species and regions, and distinctions between biologically active B_12_ and inactive B_12_ analogues remain important to clarify (Peñalver et al. 2020; Cherry et al. 2019).

Despite their promising nutrient content, there are also safety concerns related to seaweed consumption as highlighted in the literature (Cherry et al. 2019). Seaweeds tend to concentrate certain elements from their environment, including iodine and potentially toxic elements such as arsenic and other heavy metals (Lee et al. 2025; Leandro et al. 2020; Banach et al. 2020). While iodine is an essential nutrient required for thyroid hormone synthesis, very high iodine concentrations, particularly in certain brown seaweeds, can pose a risk of excessive intake (Lee et al. 2025; EFSA Panel on Contaminants in the Food Chain (CONTAM) 2014). Likewise, heavy metals can accumulate in seaweeds at levels influenced by environmental conditions, necessitating careful assessment of their safety for human consumption (Lee et al. 2025; Banach et al. 2020).

Processing methods such as blanching, boiling, or soaking have been shown to reduce the content of soluble compounds, including iodine, without substantially diminishing other nutrients, suggesting practical strategies to mitigate potential risks while preserving nutritional benefits (Ho and Redan 2022). Understanding how cultivation (wild versus farmed) and post‐harvest treatment impact both nutrient and contaminant profiles is therefore essential for evaluating the suitability of seaweeds as food ingredients in sustainable diets.

In this study, we have analyzed a range of wild and farmed Scottish seaweeds for their mineral and vitamin B_12_ content and assessed their safety aspects through measurement of heavy metals and iodine. We have also investigated the effect of blanching on iodine levels, with the aim of determining whether these seaweeds could represent valuable and safe sources of micronutrients for human diets, particularly within the context of sustainable and plant‐based nutrition.

## 2 Materials and Methods

### 2.1 Sampling Strategy and Sample Collection

Eleven seaweed species ( *A. esculenta* , *A. nodosum* , *F. serratus* , *F. spiralis* , *F. vesiculosus* , *H. elongata* , *L. digitata* , *P. palmata* , *P. canaliculata* , *Porphyra* sp., and *S. latissima*) were included in the study. Samples were obtained from New Wave Foods Ltd., Aberdeen, Scotland (note that due to the difficulty in distinguishing between *Porphyra* species, only the genus is given). These species were investigated as they have either been used previously in food applications or display nutritional characteristics suggesting their suitability for such uses. 71 seaweed samples were wild harvested between July 2016 and May 2022 from the north and northeast coast of mainland Scotland (around Caithness). Sampling locations were distributed across 16 sites along approximately 50 miles of coastline, from Brims Ness to Old Wick. Multiple (up to seven individual samples) of the same seaweed species were analyzed in order to capture variation due to seasonal and locational differences at harvesting. Harvested seaweeds were rinsed with freshwater and dried on stainless‐steel racks in drying chambers at ~40°C for 36 h under continuous airflow and controlled humidity. Dried material was hand‐cut, sealed in high‐density polyethene bags, and stored at 10°C–20°C in the absence of air and light. For subsequent analysis, samples were milled to a fine powder (using a household blender) and stored in amber glass bottles at room temperature until required. In addition, farmed seaweed samples were obtained from two seaweed farms on the west coast of Scotland (south of Oban). Details on the species and sampling locations are provided in Table 1.

**TABLE 1:** Seaweed samples used in the current study.

Scientific name	Wild/farmed	Location	Number of samples
*Alaria esculenta*	Wild	N	7
*Alaria esculenta*	Farmed	W	2
*Ascophyllum nodosum*	Wild	N	7
*Fucus serratus*	Wild	N	7
*Fucus spiralis*	Wild	N	7
*Fucus vesiculosus*	Wild	N	7
*Himanthalia elongata*	Wild	N	7
*Laminaria digitata*	Wild	N	7
*Palmaria palmata*	Wild	N	7
*Pelvetia canaliculata*	Wild	N	7
*Porphyra* sp.	Wild	N	1
*Saccharina latissima*	Wild	N	3
*Saccharina latissima*	Farmed	W	2

Abbreviations: *N*, north coast of Scotland; W, west coast of Scotland (south of Oban).

Additionally, wet whole‐leaf seaweed samples from three species ( *A. esculenta* , *S. latissima*, and *F. serratus* ) harvested on the same day from the same location on the north coast of Scotland were also obtained exclusively for the iodine blanching studies.

### 2.2 Blanching/Hydrothermal Treatment of Wet Seaweeds

Wet seaweed samples were cut into pieces and mixed to give a uniform batch of each sample. Approximately 100 g aliquots were weighed out and blanched in a water bath at different temperatures (30°C, 45°C, 60°C, or 80°C) for 5 min. Between each blanching, the water bath was thoroughly washed and cleaned before the addition of the next sample. Once blanched, samples were drip‐dried over a sink for 10 min before being stored in sealed bags at −70°C.

### 2.3 Freeze‐Drying of Wet Seaweeds

Samples were thawed from −70°C and dried on paper towels to remove excess moisture before being weighed into containers. These were covered with Miracloth (Sigma‐Aldrich, Darmstadt, Germany) secured using elastic bands and then placed in a freeze dryer and left until the temperature reached approximately −21°C (around 6 days). The samples were then reweighed to determine water loss and stored at −70°C until powdering for analysis. To obtain powdered material, seaweed samples (20–25 g) were pre‐frozen using liquid nitrogen in a cryogenic freezer mill (Freezer mill Spex CertiPrep 6800, UK) and then ground twice in 2 min cycles at the rate of 12 turns/s. Samples were then stored at −70°C until required.

### 2.4 Sample Digestion and Mineral and Heavy Metal Analysis

Powdered seaweed samples were digested using a microwave accelerated reaction system (MARS 6 digestion system (CEM, Matthews, USA)) in which 0.2 g of material was treated with high‐purity nitric acid and hydrochloric acid (Sigma Aldrich). Gold was added at a concentration of 0.4 mg/L to stabilize Hg through amalgamation and to preserve the Hg signal during the inductively coupled plasma–mass spectrometry (ICP‐MS) analysis (Agilent 7700X ICP‐MS, USA). Elemental analysis was then performed using the ICP‐MS instrument equipped with a MicroMist nebulizer for sample introduction and He as the collision cell gas. An intelligent wash method (continuous washing until near zero reading with Hg) was used between samples to reduce any Hg contamination from the previous sample. Erbium (Er) was used as an internal standard for the sample. Each analysis had a run time of 3 min, with the data acquisition set to 1 point, 5 replicates, and 100 sweeps per replicate. The ICP‐MS analysis of seaweed samples detected levels of ^23^Na, ^24^Mg, ^31^P, ^39^K, ^40^Ca, ^55^Mn, ^56^Fe, ^59^Co, ^63^Cu, ^66^Zn, ^75^As, ^78^Se, ^98^Mo, ^127^I, ^111^Cd, ^202^Hg, and ^208^Pb. Sample decomposition was carried out using a matrix consisting of nitric acid, hydrochloric acid, and 200 ng/mL of Au in deionized water. Method accuracy was monitored using ERM‐CE278k (Mussel tissue) and ERM‐CD200 (Seaweed, Bladderwrack) certified reference materials (Merck Life Sciences, UK). The average recoveries for the Mussel and Seaweed CRMs for total arsenic (As), cadmium (Cd), mercury (Hg), and lead (Pb) were very good with recoveries of 100%–101%, 89%–96%, 86%–110%, and 80%–93%, respectively.

For determination of seaweed iodine levels, samples were digested at 90°C in 5% Tetramethylammonium hydroxide (TMAH) (≥ 97%, Sigma Aldrich, Gillingham, Dorset, UK) for 3 h and then cooled and centrifuged at 2500 ***g*** for 5 min, prior to running on the ICP‐MS in standard mode using external calibration. TMAH was diluted to 5% using ultrapure water (18.2 MΩ cm, ELGA PureFlex, UK). A stock standard solution was gravimetrically prepared in‐house from high purity potassium iodide (+99.99%, Thermo Fisher Scientific, UK) in 5% TMAH. Calibration standards were prepared by serial dilution of this stock, using 5% TMAH, and a Tellurium internal standard also added at the same level as in the samples (150 ng/mL final concentration). Method accuracy was monitored using ERM‐BD150 Skimmed Milk Powder certified reference material (Merck Life Sciences, UK). Average recovery of iodine from the skimmed milk was 103.7%. Limits of detection (LOD) and quantification (LOQ) were defined and determined as the minimum detectable amounts of analyte with a signal‐to‐noise ratio of 3:1 and 10:1, respectively (Miller and Miller 2000). The LOD for iodine was calculated as 13.5 mg/kg dw and the LOQ was 41.0 mg/kg dw.

### 2.5 Measurement of Vitamin B_12_ Content

Dried seaweeds (~0.2 g) were accurately weighed and assayed for vitamin B_12_ using the RIDASCREEN FAST Vitamin B12 ELISA (R‐Biopharm Rhone Ltd., UK) assay kit as per the manufacturer's instructions using the method described for “Grains and cereals”. The kit detects all four forms of vitamin B_12_ (cyano‐, hydroxo‐, methyl‐ and adenosyl‐cobalamin) and samples were assayed in triplicate, at either 1 in 10 or 1 in 25 dilutions.

### 2.6 Assessing Potential Contribution of Seaweed Micronutrients to Dietary Intakes and Health Risk Assessment Heavy Metals

Estimated average seaweed consumption for adults in Asian countries can range from 4 g/d in Japan to 8.5 g/d for South Korea (Roleda et al. 2019). However, as seaweed consumption data for Europeans is currently not available, the assessed health benefits or health risks were calculated using a single serving of 5 g (dw), once a week, and an average body weight (BW) of 60 kg for an adult. To estimate health benefits, the amount of micronutrient in the serving was compared as a percentage of the reference nutrient intake (RNI) for that nutrient based on the following RNIs: 1600 mg/d Na, 300 mg/d Mg (male), 550 mg/d P, 3500 mg/d K, 700 mg/d Ca, 8.7 mg/d Fe (males), 1200 mg/d Cu, 9500 mg/d Zn, 75 mg/d Se and Adequate Intakes (AI) of 3 mg/d Mn and 45 mg/d Mo (EFSA 2013; Institute of Medicine 2001) while there is currently no RNI/AI set for Co.

The margin of exposure (MOE) was used for the health risk assessment of inorganic As (iAs) and Pb. The MOE for iAs calculation was based on the estimated benchmark dose (BMDL_0.1_ of 0.3–8 μg/kg body weight (BW)/day), that causes a 1% increased risk of skin, lung, and bladder cancer, based on epidemiologic research (EFSA Panel on the Contaminants in the Food Chain 2009). For the iAs risk assessment, BMDL_0.1_ of 0.3 μg/kg BW/day was used as a representative point which corresponds to the lower end of the BMDL_0.1_ value range. For Pb, the MOE was based on the BMDL_0.1_ set by the European Food Safety Authority (EFSA) which was associated with a 1% extra risk for neurodevelopmental effects in children of 12 μg/L in blood, which corresponded to 0.50 μg Pb/kg BW/day (EFSA CONTAM Panel on Contaminants in the Food Chain 2010). An MOE of 10 or greater was considered sufficient (exposure ≤ 0.05 μg/kg BW/d) (EFSA CONTAM Panel on Contaminants in the Food Chain 2010). For Hg, Cd, and iodine, estimated exposure values for consumption of one 5 g portion of dried seaweed per week were compared to the Tolerable Weekly Intake (TWI) or Tolerable upper limit (UL) values for these elements.

### 2.7 Statistical Analysis

Effects of species location, season, and year on levels of seaweed constituents measured in this study were compared by one‐way or two‐way analysis of variance (ANOVA). Means were compared by post hoc tests with Tukey adjustment for multiple comparisons. Pearson correlations were calculated to assess the association between different metals and iodine or vitamin B_12_. Significance was set at *p* < 0.05.

## 3 Results

### 3.1 Micronutrient Levels in Scottish Seaweeds

#### 3.1.1 Vitamin B_12_ Levels

**FIGURE 1:**
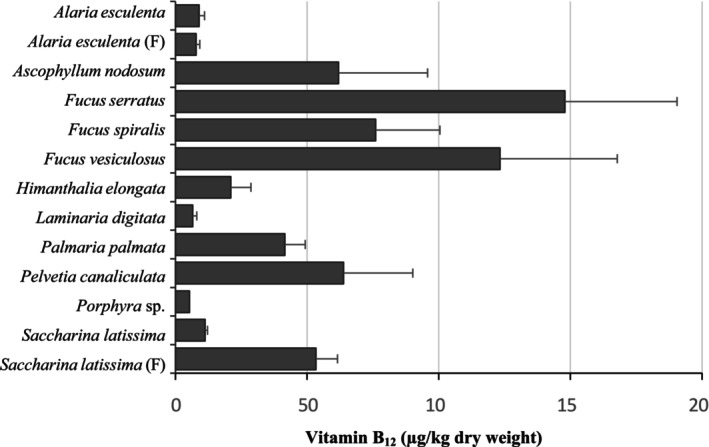
Vitamin B_12_ content (μg/kg) of different seaweed species harvested from wild and farmed (F) sources in Scotland. Values are expressed as the mean ± SEM (standard error of the mean).

All seaweed samples collected from the north and west coasts of Scotland contained detectable levels of vitamin B_12_ (Figure 1). The highest vitamin B_12_ concentrations were observed in *Fucus* species, with *F. serratus* showing a mean value of 148.0 ± 42.5 μg/kg dry weight. Elevated vitamin B_12_ levels were also measured in *A. nodosum* and *P. canaliculata* at 62.0 ± 33.8 and 63.8 ± 26.3 μg/kg, respectively (Figure 1). Vitamin B_12_ concentrations in wild and farmed *A. esculenta* did not differ markedly from each other. In contrast, farmed *S. latissima* samples from the west coast contained vitamin B_12_ levels approximately five times higher than those measured in wild samples harvested from the northwest coast (Figure 1). Substantial variability in vitamin B_12_ content was observed both between and within species, particularly among those with the highest concentration (Figure 1). For example, vitamin B_12_ levels in *F. serratus* ranged from 14.2 to 248.9 μg/kg dry weight. No clear influence of sampling month, season, or year on vitamin B_12_ levels was detected when species were considered individually (data not shown). Moreover, no correlation was found between Co content and vitamin B_12_ concentrations in the analyzed seaweed samples (data not shown).

#### 3.1.2 Potential Contribution of Seaweeds to Dietary Vitamin B_12_ Intake

**FIGURE 2:**
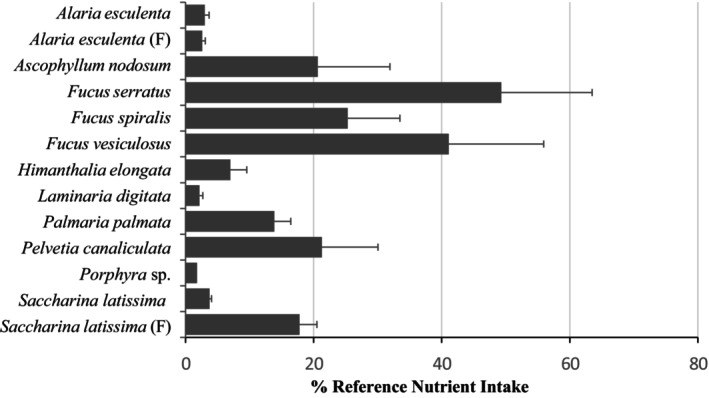
Vitamin B_12_ levels in a 5 g portion of different seaweed species from the north and west coasts of Scotland, expressed as a percentage of the reference nutrient intake (RNI) for vitamin B_12_. Values are presented as the mean ± SEM. (F), farmed seaweed.

The average vitamin B_12_ levels in a 5 g portion of each seaweed were calculated as a percentage of the RNI for this vitamin (1.5 μg/day) to examine how the consumption of different seaweed species could contribute to dietary vitamin B_12_ intake (Figure 2). Five seaweed species ( *A. nodosum* , *F. spiralis* , *F. vesiculosus* , *F. serratus* , and *P. canaliculata* ) were found to provide at least 20% of the RNI for vitamin B_12_ at this intake level. A 5 g portion of *F. serratus* would provide 49% of the RNI for vitamin B_12_ (Figure 2).

#### 3.1.3 Macromineral Content of Seaweeds

Mean macromineral contents in seaweed samples collected in this study are shown in Table 2. In all seaweed species, levels of Na and K were the most abundant, followed by levels of Mg and Ca (Table 2). The P content of seaweeds ranged from 1181 to 4945 mg/kg. Overall, levels of Na, Mg, and Ca in *Palmeria palmata* were around 16%–20% lower than average seaweed values in the other species (Table 2).

**TABLE 2:** Levels (mg/kg dry weight) of macrominerals in Scottish seaweeds.

Species	Na	Mg	P	K	Ca
*Alaria esculenta* (*n* = 7)	33,410	7955	4945	55,242	10,803
	(1329)	(360)	(666)	(3039)	(386)
*Alaria esculenta* (F) (*n* = 2)	28,400	7559	4652	56,370	10,280
	(1563)	(465)	(1370)	(4410)	(1383)
*Ascophyllum nodosum* (*n* = 7)	34,964	8234	1289	28,946	10,798
	(803)	(379)	(105)	(929)	(121)
*Fucus serratus* (*n* = 7)	32,222	7426	3120	42,142	11,618
	(1282)	(415)	(275)	(1924)	(531)
*Fucus spiralis* (*n* = 7)	32,595	7790	1651	43,303	12,509
	(1144)	(377)	(135)	(4479)	(1087)
*Fucus vesiculosus* (*n* = 7)	26,898	6540	1860	38,274	10,469
	(1700)	(261)	(233)	(4381)	(563)
*Himanthalia elongata* (*n* = 7)	53,183	9600	2108	87,118	8243
	(378)	(248)	(209)	(2792)	(128)
*Laminaria digitata* (*n* = 7)	44,027	8884	3359	65,387	12,321
	(1886)	(523)	(301)	(3600)	(227)
*Palmaria palmata* (*n* = 7)	5964	1466	3591	68,133	1595
	(565)	(78)	(215)	(1448)	(174)
*Pelvetia canaliculata* (*n* = 7)	33,816	8639	1289	31,020	10,400
	(671)	(436)	(37)	(1584)	(341)
*Porphyra* sp. (*n* = 1)	32,111	8013	1181	31,721	9629
*Saccharina latissima* (*n* = 7)	32,930	7678	2129	48,253	11,038
	(1217)	(724)	(99)	(2377)	(696)
*Saccharina latissima* (F) (*n* = 2)	27,356	6700	2891	122,692	8881
	(2787)	(1162)	(127)	(4767)	(264)

*Note:* Values are presented as means, with the SEM shown in parentheses. (F) Farmed seaweed.

#### 3.1.4 Trace Element Levels in Seaweeds (Mn, Fe Co, Cu, Zn, Se, and Mo)

Levels of trace elements, including Mn, Fe Co, Cu, Zn, Se, and Mo, were measured in all samples of seaweed collected in this study and are shown in Table 3. All three *Fucus* species had the highest levels of Mn, which ranged from 5 to 11 times the average amount for the other species (Table 3). Levels of Fe were highest in the two farmed species ( *Alaria esculenta* and *Saccharina latissima*), where levels were around 9–15 times the Fe levels in the other wild seaweed species. Farmed seaweeds showed lower Co levels compared with the wild harvested versions, with levels being around 5–22 times lower (Table 3). Levels of the other trace elements (Cu, Zn, Se, and Mo) showed some variation between seaweed species but, in general, were low in content (Table 3).

**TABLE 3:** Levels of trace elements in selected Scottish seaweeds.

Species	Mn (mg/kg)	Fe (mg/kg)	Co (mg/kg)	Cu (mg/kg)	Zn (mg/kg)	Se (mg/kg)	Mo (mg/kg)
*Alaria esculenta* (*n* = 7)	4985 (376)	94.2 (42.8)	14.9 (4.8)	635.6 (54.4)	43.4 (3.3)	69.5 (7.7)	127.8 (9.5)
*Alaria esculenta* (F) (*n* = 2)	42,233 (29,829)	1446 (1165)	587 (452)	1687 (611)	37.5 (9.9)	162.2 (86.1)	183.3 (22.7)
*Ascophyllum nodosum* (*n* = 7)	15,267 (630)	60.0 (5.3)	244.3 (23.2)	336.3 (22.5)	25.7 (1.6)	23.3 (2.2)	733.6 (22.3)
*Fucus serratus* (*n* = 7)	146,543 (14,583)	102.4 (12.8)	621.1 (61.7)	1018.5 (68.1)	37.0 (2.6)	79.1 (7.5)	161.6 (10.5)
*Fucus spiralis* (*n* = 7)	59,824 (11,793)	145.6 (30.6)	532.4 (122.8)	727.4 (101.0)	23.5 (2.7)	50.5 (3.2)	159.8 (8.5)
*Fucus vesiculosus* (*n* = 7)	84,360 (7913)	93.4 (18.3)	331.5 (65.0)	669.2 (62.1)	21.0 (2.4)	36.2 (5.2)	139.9 (8.3)
*Himanthalia elongata* (*n* = 7)	13,192 (994)	13.2 (1.2)	166.6 (27.8)	375.5 (39.7)	28.1 (2.3)	23.2 (3.9)	119.7 (10.0)
*Laminaria digitata* (*n* = 7)	3202 (178)	51.1 (9.1)	191.6 (48.2)	594.7 (27.2)	44.6 (5.4)	36.4 (4.6)	130.1 (8.8)
*Palmaria palmata* (*n* = 7)	6428 (245)	187.0 (47.0)	212.4 (43.5)	1981.3 (128.6)	22.6 (1.7)	158.6 (24.5)	276.3 (16.8)
*Pelvetia canaliculata* (*n* = 7)	12,851 (1395)	204.0 (38.1)	1071.7 (268.0)	608.8 (88.6)	14.5 (1.6)	36.4 (5.0)	280.3 (30.3)
*Porphyra* sp. (*n* = 1)	7637	61.6	258.6	439.9	13.6	28.4	249.5
*Saccharina latissima* (*n* = 7)	2474 (162)	20.6 (4.4)	66.4 (12.1)	275.7 (21.0)	11.2 (0.9)	31.6 (9.5)	122.5 (7.3)
*Saccharina latissima* (F) (*n* = 2)	25,603 (1734)	872.3 (20.4)	348.8 (83.4)	1342.7 (357.5)	39.6 (12.8)	58.2 (9.5)	233.5 (1.0)

*Note:* Values are presented as means, with the SEM shown in parentheses. (F) Farmed seaweed.

#### 3.1.5 Assessing the Contribution of Seaweed‐Derived Minerals (Macrominerals and Trace Elements) to Dietary Intakes

The calculated %RNI values within a 5 g seaweed portion obtained for Na and Mg showed that levels of these elements were at least 10% of RNI across all seaweed species except *Palmaria palmata. H. elongata* had the highest contributions of these minerals (15.9% RNI for Mg and 16.6% RNI for Na) whereas *P. palmata* contained levels of Mg and Na equating to around 2% of RNI. Levels of K in farmed *S. latissima* were the highest at 18% of the RNI. Levels of P and Ca within a 5 g portion of each of the seaweed species were relatively low and averaged 2% and 7% respectively, of their RNIs. For a 5 g portion/d, the two farmed species, *A. esculenta* and *S*. *latissima* was calculated to contribute 83% and 50%, respectively, to the RNI for Fe. The three *Fucus* species showed the highest Mn and so contribution to AI, with *F. serratus* providing 24% and *F. vesiculosus* providing 14% of the AI in a 5 g portion. Levels of Cu, Zn, Se and Mo in a 5 g portion of seaweed were all below 5% of their respective RNIs or AIs.

#### 3.1.6 Iodine Content in Seaweeds

**FIGURE 3:**
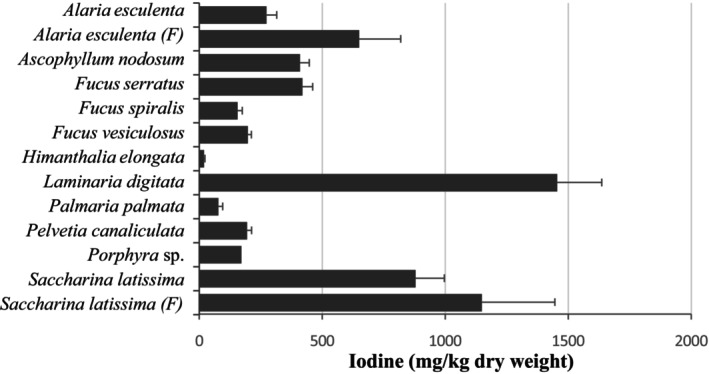
Iodine content (mg/kg dry weight) of wild and farmed seaweeds harvested in Scotland. Values represent the means ± SEM. (F), farmed seaweed.

Seaweed I content varied widely across the different seaweed species analyzed (Figure 3). Among all species, *L. digitata* contained the highest I content with a mean value of 1455 ± 181 mg/kg dry weight. Other seaweeds, including *S.* *latissima* and farmed *A. esculenta* , also showed high I concentrations (Figure 3).

#### 3.1.7 Health Risk Assessment of Iodine Content in Seaweeds

**FIGURE 4:**
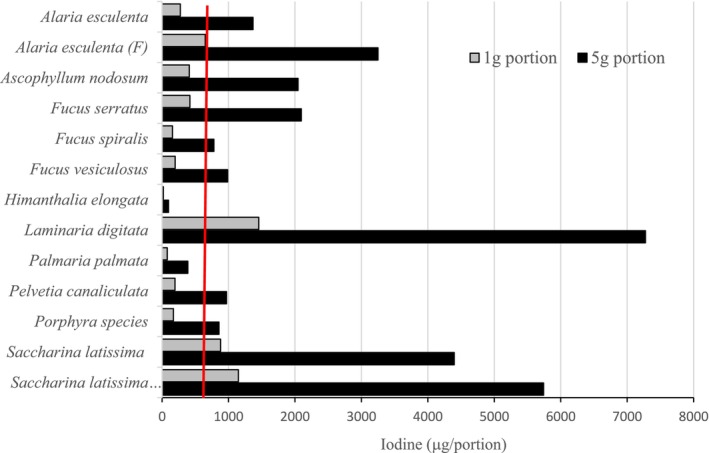
Iodine content (μg/portion) of different seaweeds at 1 and 5 g portion sizes in relation to the tolerable upper intake level (UL) for iodine. The red line represents the European UL for iodine (600 μg/day). Black columns indicate a 5 g portion, and gray columns indicate a 1 g portion.

There are limited data on habitual seaweed intake in European countries, so portion sizes of 1 and 5 g of each seaweed were assessed and compared with the Upper Limit (UL) of 600 μg/day for I in adult Europeans (EFSA 2006). A 5 g portion of most seaweed species were estimated to exceed the UL for iodine, except *Himanthala elongata* and *Palmeria palmata* (Figure 4). Indeed, levels in *Laminaria* were extremely high in that even if only one 5 g portion was consumed per week, it would still equate to an intake of 1040 μg/day of iodine. Even a 1 g portion of *Saccharina latissima*, *Alaria esculenta* , and *Laminaria digitata* would exceed the UL for iodine (Figure 4).

### 3.2 Heavy Metal Content of Seaweeds

Heavy metal levels within the seaweeds in the current study are outlined in Table 4. Seaweed tAs levels were the highest out of the four heavy metals assessed within the study (around 50–100 times average levels of Cd or Pb). Levels of tAs were generally similar within all species (averaging 40 mg/kg dw) with the highest levels in *Laminaria digitata* being 12 times the lowest levels in *Palmeria palmata* (Table 4), The highest levels of Cd were generally found in *Himanthalia elongata* and *Fucus serratus* seaweeds (averaging 1.8 and 1.5 mg/kg dw, respectively) (Table 4).

**TABLE 4:** Levels of heavy metals in seaweeds.

Species	tAs (mg/kg dw)	Cd (μg/kg dw)	Hg (μg/kg dw)	Pb (μg/kg dw)
*Alaria esculenta* (*n* = 7)	47.3 ± 4.1	1061.2 ± 103.0	0.6 ± 0.3	113.6 ± 50.3
*Alaria esculenta* (F) (*n* = 2)	46.1 ± 7.1	1359.3 ± 100.4	5.2 ± 0.5	1484.5 ± 1185.2
*Ascophyllum nodosum* (*n* = 7)	22.2 ± 1.2	262.2 ± 22.0	7.4 ± 2.8	111.8 ± 22.7
*Fucus serratus* (*n* = 7)	49.5 ± 2.0	1498.3 ± 108.6	3.1 ± 1.3	294.4 ± 39.2
*Fucus spiralis* (*n* = 7)	29.4 ± 1.5	781.4 ± 86.5	3.7 ± 1.5	139.3 ± 17.2
*Fucus vesiculosus* (*n* = 7)	37.4 ± 2.7	838.2 ± 91.5	6.5 ± 2.2	208.7 ± 53.8
*Himanthalia elongata* (*n* = 7)	24.2 ± 2.2	1824.0 ± 320.2	0.5 ± 0.5	23.4 ± 4.4
*Laminaria digitata* (*n* = 7)	99.0 ± 4.0	92.7 ± 12.2	4.2 ± 1.6	34.9 ± 6.7
*Palmaria palmata* (*n* = 7)	8.0 ± 0.5	216.1 ± 33.0	1.4 ± 0.7	239.2 ± 99.0
*Pelvetia canaliculata* (*n* = 7)	24.2 ± 1.4	213.5 ± 19.6	31.5 ± 5.5	230.9 ± 40.8
*Porphyra* sp. (*n* = 1)	23.5	285.7	35.9	63.6
*Saccharina latissima* (*n* = 3)	61.2 ± 3.0	733.9 ± 140.0	1.9 ± 1.1	29.7 ± 4.4
*Saccharina latissima* (F) (*n* = 2)	46.4 ± 13.0	231.2 ± 119.9	19.7 ± 0.5	922.3 ± 108.3

*Note:* Values represent the means ± SEM. (F), farmed seaweed; (dw), dry weight; (tAs) total As.

Levels of Pb were generally low and quite variable in some species. Pb appeared higher in farmed‐grown *Alaria esculenta* and *Saccharina latissima* than in the wild harvested forms, although sample size was limited in these cases (Table 4). The highest levels of Hg were found in *Pelvetia canaliculata* and *Porphyria sp* (Table 4), however, overall levels of Hg were low. Individual seaweed species usually did not contain elevated levels of multiple heavy metals.

#### 3.2.1 Health Risk Assessment of Heavy Metals in Seaweeds

Currently, of the four heavy metals analyzed here, only Cd levels in seaweed are governed by European regulation where permitted levels are 3.0 mg/kg wet weight (EC Commission 2014). In European adults, the average exposure to Cd is already near to or above the TWI (Tolerable Weekly Intake) for Cd at 2.5 μg/kg BW/wk (EC Commission 2014). In the current study, average Cd levels in *Himanthalia elongata* within a single serving of 5 g/wk. was calculated to contribute to a Cd weekly intake of 0.152 μg/kg BW, which is equivalent to 6% of the TWI. Similarly, *Fucus serratus* , and *Alaria esculenta* would both contribute 5% of the TWI for Cd. These levels would therefore be inconsequential compared to other sources.

Regarding the potential toxic effects of As, as only total arsenic (tAs) content was measured, an accurate risk assessment cannot be carried out as levels of iAs (the most toxic form) were not assessed. However, an assessment of risk was approximated using data from previous studies determining the relative proportions of iAs forms to organic forms of As within the different seaweeds (Maulvault et al. 2015; Ronan et al. 2017). These studies showed that the proportion of iAs of the tAs within seaweed species can vary but are often very low (around 1%) apart from in *Laminaria digitata* (Maulvault et al. 2015; Ronan et al. 2017; Rose et al. 2007; Taylor and Jackson 2016) and *Sargassum* species (Kim et al. 2024). In *L. digitata* , proportions of iAs have been shown to be up to 50% of tAs (Maulvault et al. 2015; Ronan et al. 2017; Taylor and Jackson 2016). Therefore, the value of 50% was used for *Laminaria digitata* and 1% for each of the other seaweeds as percentages of iAs of the tAs content, and the approximate risk of exposure to iAs, assuming consumption of one 5 g serving/week, was estimated. For *L. digitata* , this corresponded to an intake level of 0.589 μg iAs/kg BW/d, which, in relation to the BMDL_0.1_ of 0.3 ug/kg BW/d for iAs (EFSA Panel on the Contaminants in the Food Chain 2009), gives a margin of exposure (MOE) of 0.5, whereas the other seaweeds would give MOE values of 41–315. Therefore, consumption of *L. digitata*, but not the other seaweed species, would present a significant risk to consumer health at this consumption level. Regarding Pb toxicity, consumption of a 5 g serving/wk. of farmed *A. esculenta* and *S. latissima* (which contained the highest Pb levels) was estimated to contribute to a Pb intake of 0.018 and 0.011 μg/kg BW/d and an MOE of 28 and 46, respectively, based on the BMDL_0.1_ of 0.5 μg Pb/kg BW/d (EFSA CONTAM Panel on Contaminants in the Food Chain 2010), although levels were variable and sample number limited. Conversely, cconsumption of a 5 g serving/wk. for seaweeds containing the lowest levels of Pb ( *H. elongata* and *L. digitata* ) would contribute to Pb intakes of 0.0003 and 0.0004 μg/kg BW/d respectively (with both MOEs > 1000) and present a low risk to health. For total Hg, a TWI of 4 μg/kg BW per week has been established (EFSA CONTAM Panel on Contaminants in the Food Chain 2012), and therefore the calculated intake of total Hg from one 5 g serving/wk. in the highest Hg‐containing seaweed (*Porphyra* spp.), amounted to an intake of 0.003 μg/kg BW/wk., indicating that the total exposure to Hg from the seaweeds assessed here would be negligible.

### 3.3 Effect of Blanching on Seaweed Iodine and Heavy Metal Levels and Subsequent Risk to the Consumer

**FIGURE 5:**
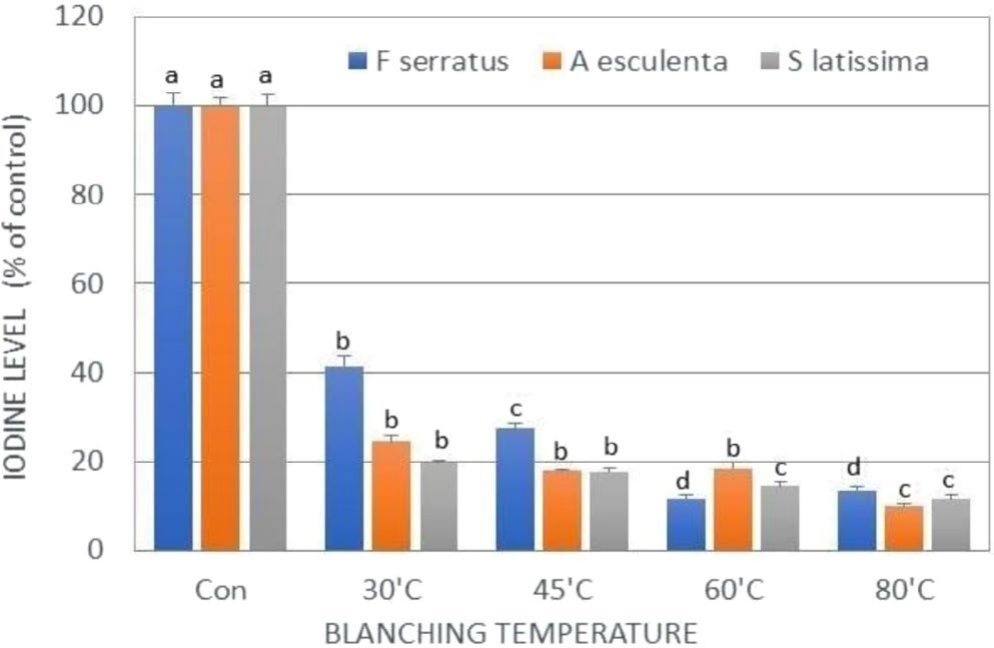
Effect of blanching different seaweed species in water at different temperatures for 5 min on iodine levels. Iodine levels are expressed as a percentage of those in unblanched controls *F. serratus* (blue columns); *A. esculenta* (orange columns); *S. latissimi* (gray columns). Statistical analysis was performed using ANOVA, and different lowercase letters above bars indicate statistically significant differences between treatments within the same seaweed species (*p* < 0.05).

Seaweeds were blanched in fresh water at different temperatures for 5 min to determine the optimum temperature capable of reducing I and heavy metal content. Four temperatures were selected: 30°C, 45°C, 60°C, and 80°C, and three seaweed species were examined (*Fucus serratus*, *Alaria esculenta*, and *Saccharina latissima*) as these seaweeds contain some of the highest levels of I of the species in the current study (see Figure 3). As shown in Figure 5, blanching at all four temperatures significantly reduced seaweed I levels in all three species by around 86%–90% of the levels present in unblanched seaweed (Figure 5). The minimum temperature that invoked the greatest loss of I in both *F. serratus* and *S. latissima* was 60°C, as no further significant losses were observed at 80°C (Figure 5). For these seaweeds, I levels were reduced to 12% and 14%, respectively, of the levels in unblanched seaweeds.

The blanching treatment of seaweed was also shown to reduce tAs levels, with both *F. serratus* and *S. latissima* exhibiting temperature‐dependent reductions in tAs levels, with the highest temperature significantly reducing levels to 52% (*p* < 0.0001) and 39% (*p* < 0.0001) respectively of the unblanched seaweeds (data not shown). However, there was no significant effect of blanching at any temperature on tAs levels in *A. esculenta* (not shown). Blanching also did not have any significant effects on the levels of the other heavy metals (Cd, Pb, Hg) within the three seaweed species assessed under the current conditions (data not shown).

## 4 Discussion

This aim of this study was to define which Scottish seaweed species could provide safe sources of nutritionally relevant levels of micronutrients for human consumption. Seaweeds are ideally suited to providing a sustainable, plant‐based source of many important dietary micronutrients (as well as macronutrients) required for optimal health and protection against disease. However, as seaweeds also accumulate potentially toxic elements, any benefits of seaweed consumption need to be assessed alongside the potential adverse effects from levels of these elements. Results from the current study show that several seaweed species could provide a plant‐based source of important dietary micronutrients, and for some species, risks to health can be reduced by processing methods such as blanching at moderate temperatures.

This study has shown that Scottish‐grown *A. nodosum* , *F. serratus* , *F. spiralis* , *F. vesiculosus* , and *P. canaliculata* (although not currently classified as a novel food) could provide dietary‐relevant levels of vitamin B_12_ (up to 50% of RNI in 5 g portion). This would be particularly relevant for population groups such as vegetarians and older adults, who experience difficulty in meeting daily vitamin B_12_ requirements (Niklewicz et al. 2024). There have been reports that vitamin B_12_ in algae might not be bioavailable in humans, which may be because some unicellular algae can contain pseudo‐cobalamin forms which are physiologically inactive (Dagnelie et al. 1991; van den Berg et al. 1991). However, within macroalgae/seaweeds, studies have shown that various forms of active cobalamin are present within seaweed (Takenaka et al. 2001) and that other studies, within both rodents and humans, suggest that this vitamin B_12_ is in a form that is bioavailable (Takenaka et al. 2001; Huang et al. 2024) although further human intervention studies are needed to determine whether seaweed consumption can reliably meet dietary B_12_ needs.

It is worth noting here that both *A. nodosum* and *F. serratus* also contained high levels of I, potentially posing a risk to health if consumed on a regular basis. However, data presented here (as well as other studies (Correia et al. 2021)) have demonstrated that I levels can be reduced by up to 90% in many different seaweed species by blanching in heated water, thereby alleviating the potential risk from I using this procedure. However, whether blanching may also reduce levels of vitamin B_12_ is not known, but heat can reduce B_12_ stability when present in foods, so this requires further investigation (Temova Rakuša et al. 2022).

The K and Mg content of most seaweeds analyzed here could contribute to RNIs, but another main benefit of seaweeds is their suitability as low salt alternatives in foods, as their higher K and Mg content (as well as Ca) in relation to Na content help replicate the flavor of salt at much lower Na levels (Murphy et al. 1981). Results here also suggest that the two farmed species, *A. esculenta* and *S*. *latissima*, contain nutritionally relevant levels of Fe, unlike the wild forms. Whether levels are specifically related to the farming process or location, as these samples were from the west coast of Scotland, or not is clear, but similar high Fe levels have been reported in seaweeds in other studies (Cherry et al. 2019). The bioavailability of the seaweed Fe has previously been determined in studies with cooked algae and shown to range between 12% and 22%, which is higher than the Fe bioavailability of 5%–12%, typically observed in those consuming vegetarian diets (Masuda et al. 2015). In terms of the potential risks to health posed by farmed *A. esculenta* and *S. latissima*, these seaweeds also contained highest levels of Pb (albeit levels were variable and sample numbers limited), which might present a greater risk to health if consumed at levels greater than 15 g/week, which may be needed in order to make a sizable contribution to Fe intakes. Levels of other minerals (e.g., Zn, Se, Cu, Mo, and Co) assessed in this study of seaweeds from the Scottish northwest coast were relatively low and would not make a meaningful contribution to dietary intakes. Furthermore, levels of Hg and Cd in the seaweeds analyzed were also low, consistent with those reported in previous studies (Sá Monteiro et al. 2019), with estimates showing that these metals do not pose a significant health risk at intake levels considered here.

The data generated here also show that I levels in both *P. palmata* and *H. elongata* are commensurate with being able to contribute to dietary I intakes, with the former seaweed containing much lower levels of Cd and possibly iAs, and therefore lower risks to health at higher consumption levels. The bioavailability of I, particularly in brown seaweeds, such as *H. elongata* , is generally considered high (31%–90%) although it can depend on the seaweed species and the chemical form of I present (Aquaron et al. 2002; Blikra et al. 2022). However, the I content in all the other seaweed species included in the study, if consumed regularly at levels > 3–20 g/wk., would be above the UL for I, posing a risk to health. Given this, as mentioned above, we have shown that processing methods such as blanching promote drastic reductions in seaweed I content such that levels would be more amenable to contributing to safe intakes of this mineral from consumption of these seaweeds. Additionally, we have shown that potential health risks can be further reduced by this process through reduction in levels of As, at least for some species in agreement with other studies (Correia et al. 2021).

This study has several limitations that should be noted. While the present study collected Scottish seaweed samples across multiple years, seasons, and locations to capture seaweed variability, the results are primarily based on samples from the northwestern coastal regions and may not reflect concentrations in the same species along other Scottish or UK coastlines. In addition, since limited data exists on seaweed consumption in the UK/EU, a portion size of 5 g/wk. was used as a proxy for assessing risk of heavy metal consumption when this might be an overestimate. Moreover, potential nutritional benefits of seaweeds, such as contributions to RNI or AI values, were calculated using an intake of 5 g per day, and this difference should be taken into account when drawing conclusions. Lastly, the assessment of heavy metal exposure and risk to health from seaweed consumption is likely an overestimation since the effects of cooking and processing, which commonly decrease levels of multiple elements, as well as bioavailability, have not been considered here.

Further research is needed to better quantify vitamin B_12_ levels in commonly consumed Scottish seaweeds and other relevant species and to identify factors (e.g., season, location etc.) influencing vitamin B_12_ levels so that levels can be maximized. Additional studies should also assess the bioavailability of micronutrients from different seaweed species to increase understanding of their contribution to consumer intakes, and to further characterize levels of iAs, the toxic form of As, across different seaweed species in order to better define the risks associated with consumption of this form of heavy metal from seaweeds. Lastly, in order to maximize the benefits of seaweed consumption while minimizing potential risks, blanching protocols should be optimized to maximize reduction in I and heavy metals, while minimizing losses of the other nutritionally relevant components (e.g., vitamin B_12_).

## 5 Conclusions

In conclusion, the current study has identified several seaweed species as sources of nutritionally relevant minerals, including K, Mg, Fe, I, and vitamins, particularly vitamin B_12_, which could provide sustainable plant‐based sources of these essential micronutrients. Furthermore, we show that a simple blanching treatment can be an effective processing method to reduce excessive I (and potentially iAs levels) in relevant seaweed species potentially allowing for increased utilization of these seaweeds in the food sector. Lastly, we have identified that farmed seaweed contains many of the same levels of micronutrients as wild seaweeds confirming that when produced at scale, seaweeds will remain a significant source of these nutrients as potential food/food ingredients.

## Author Contributions

**Ibrahim Almosa:** formal analysis, writing – original draft, writing – review and editing, investigation, methodology, visualization. **Alan A. Sneddon:** conceptualization, methodology, formal analysis, supervision, funding acquisition, writing – original draft, writing – review and editing, project administration, visualization, data curation. **Shabina Bashir:** methodology, investigation, writing – review and editing, project administration.

## Funding

This research was funded by the Scottish Government's Rural and Environment Science and Analytical Services Division (RESAS), grant RI‐B7‐05. IA was funded by a grant from the Cultural Bureau, Embassy of Saudi Arabia (SACB) for his PhD studentship.

## Conflicts of Interest

The authors declare no conflicts of interest.

## Data Availability

The data that support the findings of this study are openly available in Mendeley at https://data.mendeley.com, reference number 10.17632/sv7k3gkss6.1.
